# Selection of biophysically favorable antibody variants using a modified Flp-In CHO mammalian display platform

**DOI:** 10.3389/fbioe.2023.1170081

**Published:** 2023-05-09

**Authors:** Olli Huhtinen, Rune Salbo, Urpo Lamminmäki, Stuart Prince

**Affiliations:** ^1^ Protein and Antibody Engineering, Orion Corporation, Turku, Finland; ^2^ Department of Life Technologies, University of Turku, Turku, Finland; ^3^ MediCity Research Laboratory, University of Turku, Turku, Finland; ^4^ Institute of Biomedicine, University of Turku, Turku, Finland

**Keywords:** antibody engineering, mammalian display, Bxb1 integrase, recombinase mediated cassette exchange (RMCE), Flp-In CHO, antibody developability, biophysical characterization, next generation sequencing—NGS

## Abstract

Mammalian display enables the selection of biophysically favorable antibodies from a large IgG antibody library displayed on the plasma membrane of mammalian cells. We constructed and validated a novel mammalian display platform utilizing the commercially available Flp-In CHO cell line as a starting point. We introduced a single copy of a landing pad for Bxb1 integrase-driven recombinase-mediated cassette exchange into the FRT site of the Flp-In CHO line to facilitate the efficient single-copy integration of an antibody display cassette into the genome of the cell line. We then proceeded to demonstrate the ability of our platform to select biophysically favorable antibodies from a library of 1 × 10^6^ displayed antibodies designed to improve the biophysical properties of bococizumab *via* randomization of problematic hydrophobic surface residues of the antibody. Enrichment of bococizumab variants *via* fluorescence-activated cell sorting selections was followed by next generation sequencing and thorough characterization of biophysical properties of 10 bococizumab variants that subsequently allowed attribution of the mutations to the biophysical properties of the antibody variants. The mammalian displayed variants exhibited reduced aggregation propensity and polyreactivity, while critically retaining its target binding thereby demonstrating the utility of this valuable tool.

## Introduction

The selection of monoclonal antibodies (mAbs) from a mammalian display library is a useful tool in therapeutic antibody engineering (Akamatsu et al., 2007; Zhou et al., 2010). In this context mammalian display works by expressing a membrane-anchored full-length antibody recombinantly upon the plasma membrane of a mammalian cell that encodes that antibody thereby linking phenotype (binding and expression) to genotype (DNA sequence). Importantly this enables the screening of large repertoires of antibodies and selection of variants based on attributes such as antigen binding level and display level using fluorescence activated cell sorting (FACS). Mammalian cells have a series of protein quality-control checkpoints and mechanisms, which prevent the accumulation of misfolded, aggregation prone, non-specifically sticky or toxic polypeptides (Apaja and Lukacs, 2014; Sun and Brodsky, 2019). Dyson et al. have shown a strong correlation between the poor biophysical properties of an antibody in terms of polyreactivity and self-interaction and its low display level achieved upon mammalian HEK293 cells, that could not be replicated *via* phage display or yeast display (Dyson et al., 2020). It is this inherent property of mammalian cells that can be exploited in therapeutic antibody engineering to select biophysically desirable variants that display at a high density.

While transient transfection has been historically used for generation of mammalian cell libraries (Higuchi et al., 1997; Ho and Pastan, 2009), the field has been moving towards stable transfections, as this allows multiple rounds of enrichment without losing the antibody expression and the gene encoding it. Depending on the stable transfection strategy used, one or multiple copies of antibody genes can be integrated into the genome of the host cell, either in a site-specific manner or randomly. Highly efficient stable transfection of multiple copies of antibody genes randomly inserted into the genome has the potential to display very large libraries using various approaches including episomal vectors (Akamatsu et al., 2007; Bowers et al., 2011), transposon technology (Waldmeier et al., 2016), or viral transduction (Beerli et al., 2008; Zhang et al., 2012). However, the introduction of multiple antibody genes randomly into a single cell negatively impacts the genotype to phenotype coupling potentially leading to display of erroneous combinations of heavy and light chains that can result in the isolation of many passenger antibody genes, thereby reducing the rate of enrichment of specific clones (Parthiban et al., 2019). This problem can be alleviated by making a pseudo-monoclonal library through careful titration of the viral particles or DNA used for transfection in such a manner to have an average of one transfected cell displaying only one protein variant (Valldorf et al., 2022). This approach however compromises between the library size and single cell insertion. Conversely, random integration of antibody genes also has the downside of potential variation in transcription levels due to dependence of transcriptional activity at the integration locus. Thus, site-specific integration of antibody genes has the advantage of transcriptional normalization, which enables selection based solely on the properties of the displayed antibody.

Several site-specific integration strategies for mammalian cells have been presented with varying success. The Flp/FRT system using FLP recombinase for genomic integration of exogenous DNA into mammalian cells has been used for more than 3 decades (O’Gorman et al., 1991) and has been used successfully to generate mAb expressing cell lines (Zhang et al., 2015; Chen et al., 2016). However, the success of the Flp/FRT system in creating mammalian display libraries has been limited (Zhou et al., 2010; Parthiban et al., 2019) due to integration being reversible and inefficient; FRT sites remain in the genome where the reaction, in which integrated DNA is excised, is favored (Brown et al., 2011). Furthermore, sometimes (at a rate of up to 8%, see discussion), the Flp/FRT system can introduce more than one transgene consecutively. More recently, site-specific integration of antibody genes into mammalian cells *via* nuclease directed integration has been successfully demonstrated by the McCafferty group (Parthiban et al., 2019; Dyson et al., 2020) who used transcription activator-like effector nucleases (TALEN) or CRISPR/Cas9 to introduce a double-stranded break in the AAVS1 locus in the genome of mammalian cells. By utilizing the cell’s own DNA repair mechanism, they were able to introduce an antibody expression cassette into the genome by having homology arms flanking the expression cassette leading to the creation of libraries containing millions of stable cells each displaying a single antibody on their plasma membrane.

An attractive alternative to nuclease directed integration is the use of large serine integrases, that can be more specific and efficient than meganucleases, zinc finger nucleases, TALEN or CRISPR/Cas9 (Olorunniji et al., 2016). In contrast to FLP recombinase, which is a tyrosine integrase, the reaction catalyzed by serine integrases are irreversible (Brown et al., 2011). Out of 15 commonly used serine integrases, Bxb1 integrase has been identified as the most accurate and efficient for integration of DNA into human genome (Xu et al., 2013). Several research groups have demonstrated the success of using Bxb1 integrase for mammalian cell engineering by integrating a landing pad for Bxb1 integrase mediated integration into the genome of mammalian cells (Duportet et al., 2014; Inniss et al., 2017; Matreyek et al., 2017; 2019; Gaidukov et al., 2018). The landing pad cell lines are generated by integrating the landing pad into a known locus by nuclease directed integration or randomly by lentiviral transduction.

Here we present a fast and simple way of generating a cell line suitable for mammalian display libraries by integrating a Bxb1 landing pad into the genome of an off-patent, commercially available Flp-In Chinese hamster ovary (CHO) cell line. By using the Flp/FRT system as a starting point, we were able to rapidly generate robust adherent and suspension-adapted single copy Bxb1 landing pad CHO cell lines without the need for thorough cell line stability studies. We not only optimized the use of the platform for generating whole antibody display libraries containing up to tens of millions of variants, but also validated the ability of this platform to differentiate antibodies based on their biophysical properties by mutating an antibody with biophysical liabilities and using this platform to enrich variants with high affinity and high display levels that were subsequently shown to contain improved biophysical properties upon characterization.

## Results

### Mammalian display design

**FIGURE 1:**
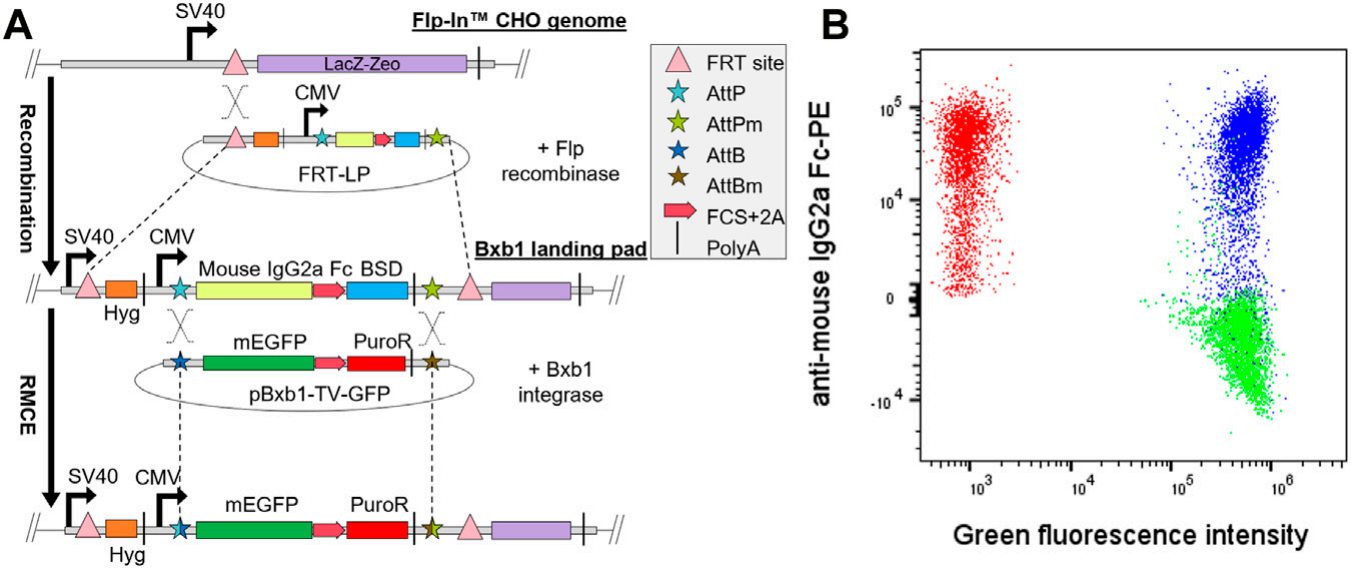
Generation of single copy Bxb1 landing pad (LP) cell line. **(A)** Bxb1 LP was first integrated into Flp-In CHO cells by co-transfection of the LP in pcDNA5/FRT vector and Flp recombinase expression vector (pOG44). Isolated LP clones were then co-transfected with pBxb1-TV-GFP and pBxb1-EV to screen for clones with only one copy of LP. **(B)** After drug selection, the expression of mEGFP and LP surface tag (mouse IgG2a Fc) was analyzed by flow cytometry. One of twelve analyzed clones expressed both GFP and mouse IgG2a Fc (blue) indicating multiple copies of LPs. Eleven of twelve analyzed clones expressed only mEGFP and thus contained one copy of the LP (green). Non-transfected LP cells were used as a control (red). FCS, furin cleavage site.

To achieve efficient integration of one copy of an antibody gene into the genome of a host cell line, we designed a landing pad (LP) for recombinase mediated cassette exchange (RMCE) utilizing Bxb1 integrase. RMCE requires three components to work: 1) recombinase-specific attachment sites (AttP) contained in a LP, located in a highly active locus of the host cell line; 2) targeting vector (TV) with corresponding recombinase-specific attachment sites (AttB) flanking the expression cassette and 3) active integrase translocated into the nucleus of the cell. We designed the Bxb1 LP to contain the cytomegalovirus (CMV) promoter driving a bicistronic expression cassette, which was flanked by Bxb1 AttP and AttPm recombinase sites (Figure 1A). The Bxb1 AttPm is a mutated version of the AttP site, where the central dinucleotide GT is mutated to GA to control the directionality of the RMCE reaction (Ghosh et al., 2003; Inniss et al., 2017). The expression cassette of the LP consisted of DNA encoding mouse IgG2a Fc fused to the transmembrane (TM) domain from platelet derived growth factor receptor beta (PDGFRβ), which was used as a surface marker.

Blasticidin S deaminase (BSD) was specifically placed as the last gene of the bicistronic expression cassette to ensure maintenance of promoter activity through constant selective pressure. Since this construct would result in a single transcript with multiple genes we utilized self-cleaving 2A peptides (de Felipe et al., 2006) and furin cleavage sites (Fang et al., 2007; Chng et al., 2015) between consecutive gene products to enable the expression of several polypeptides from the same mRNA molecule.

As the second component of the RMCE, we designed bi- and tricistronic, promoterless Bxb1 targeting vectors (pBxb1-TV) that allow the integration of the expression cassette into the LP by RMCE. The bicistronic pBxb1-TV-GFP consisted of DNA encoding membrane-anchored Enhanced Green Fluorescence Protein (mEGFP) i.e., EGFP fused to the TM domain of CD8, and puromycin-N-acetyltransferase (puroR) gene, separated by a furin cleavage site and P2A peptide. The whole expression cassette was flanked by corresponding AttB and AttBm recombinase sites to enable the unidirectional RMCE into the AttP and AttPm sites in the LP. Since the pBxb1-TV was left promoterless, only cells with successful integration of the pBxb1-TV expression cassette into the LP would have the CMV promoter driving the whole expression cassette and thus survive the puromycin selection.

The final component of our RMCE system was the Bxb1 integrase translocated to the nucleus of the cell. We designed a Bxb1 expression vector (pBxb1-EV) to be driven by the CMV promoter and containing the SV40 nuclear localization signal and HA-tag in the N-terminus of the protein. Integration of a target gene into the LP occurs by co-transfecting the pBxb1-TV and pBxb1-EV into cells which contain the Bxb1 LP. By using the pBxb1-TV-GFP, we found the optimal pBxb1-EV to pBxb1-TV ratio to be 1:4 for our suspension adapted mammalian display platform (Supplementary Figure S1).

### Generation of the mammalian display cell line

The commercially available Flp-In system has been used for site-specific gene integration in mammalian cells for more than 3 decades and is easy to use, however due to poor recombination rates and reversible reactions this system required modification prior to use for mammalian display (O’Gorman et al., 1991; Matreyek et al., 2017). As CHO cells are the most often used cell line for the manufacturing of therapeutic antibodies (Dhara et al., 2018), we chose CHO cells as the host cell line for our mammalian display to enable the selection of antibodies as close to the final therapeutic molecule as possible. A commercial Flp-In CHO line containing a single copy of a Flp recombinase target (FRT) site in a transcriptionally active site was targeted with a landing pad for Bxb1 integrase driven RMCE to increase the recombination efficiency and fidelity suitable for display of antibody libraries.

The pcDNA5/FRT-LP was co-transfected into the Flp-In CHO cell line with the pOG44 plasmid encoding Flp recombinase to integrate the Bxb1 LP into the genome (Figure 1A). To verify that only one copy of the Bxb1 LP was integrated into the cell line, selected clones were co-transfected with pBxb1-EV and pBxb1-TV-GFP. Staining of cells for IgG2a Fc detection together with monitoring of mEGFP allowed identification of clones containing only IgG2a Fc expression that would indicate the integration of only a single copy of Bxb1 LP while clones co-expressing both IgG2a Fc and mEGFP would have required two or more integrated copies of Bxb1 LP and would therefore not be appropriate for use in mammalian display (Figure 1B). A clone expressing only IgG2a Fc was subsequently adapted to serum-free suspension culture to allow for increased cell density required for display of higher diversity libraries in smaller volumes. By using the commercially available Flp-In CHO cell line as a starting point, we were able to create a robust mammalian display CHO cell line with a single Bxb1 LP in a matter of weeks.

To further verify the high fidelity of the Bxb1 RMCE system, CHO-LP cells were co-transfected with a mixture of two or three pBxb1-TV vectors encoding different fluorescent proteins along with pBxb1-EV. A flow cytometry analysis after 10 days in drug selection showed 99.1% of the cells expressing only one type of fluorescent protein with less than 0.4% of the cells expressing more than one fluorescent protein, indicating efficient single copy integration of the fluorescent protein genes and a very low off-target integration rate (Supplementary Figure S2).

### Validation of mammalian display using bococizumab

To validate that our mammalian display platform was capable of differentiating antibody variants with varying biophysical properties, we sought to mutagenize an antibody with bad biophysical properties. Bococizumab is an anti-proprotein convertase substilisin/kexin type 9 (PCSK9) antibody developed for reducing LDL cholesterol by Pfizer (Liang et al., 2012). In Phase III clinical trials almost 50% of the patients that were administered bococizumab developed anti-drug antibodies (ADA) within a year of administration (Ridker et al., 2017), consequently leading to termination of the clinical program (Sun and Benet, 2020). As described by Jain et al., bococizumab has multiple red flags amongst its biophysical properties, most importantly showing high polyreactivity and self-aggregation propensities (Jain et al., 2017). As the formation of large non-denatured aggregates can trigger a highly potent antibody response *in vivo* even in the absence of T-cells (Rosenberg, 2006), the poor biophysical properties of bococizumab likely played a role in the generation of ADAs in patients and consequent failure of the clinical program.

**FIGURE 2:**
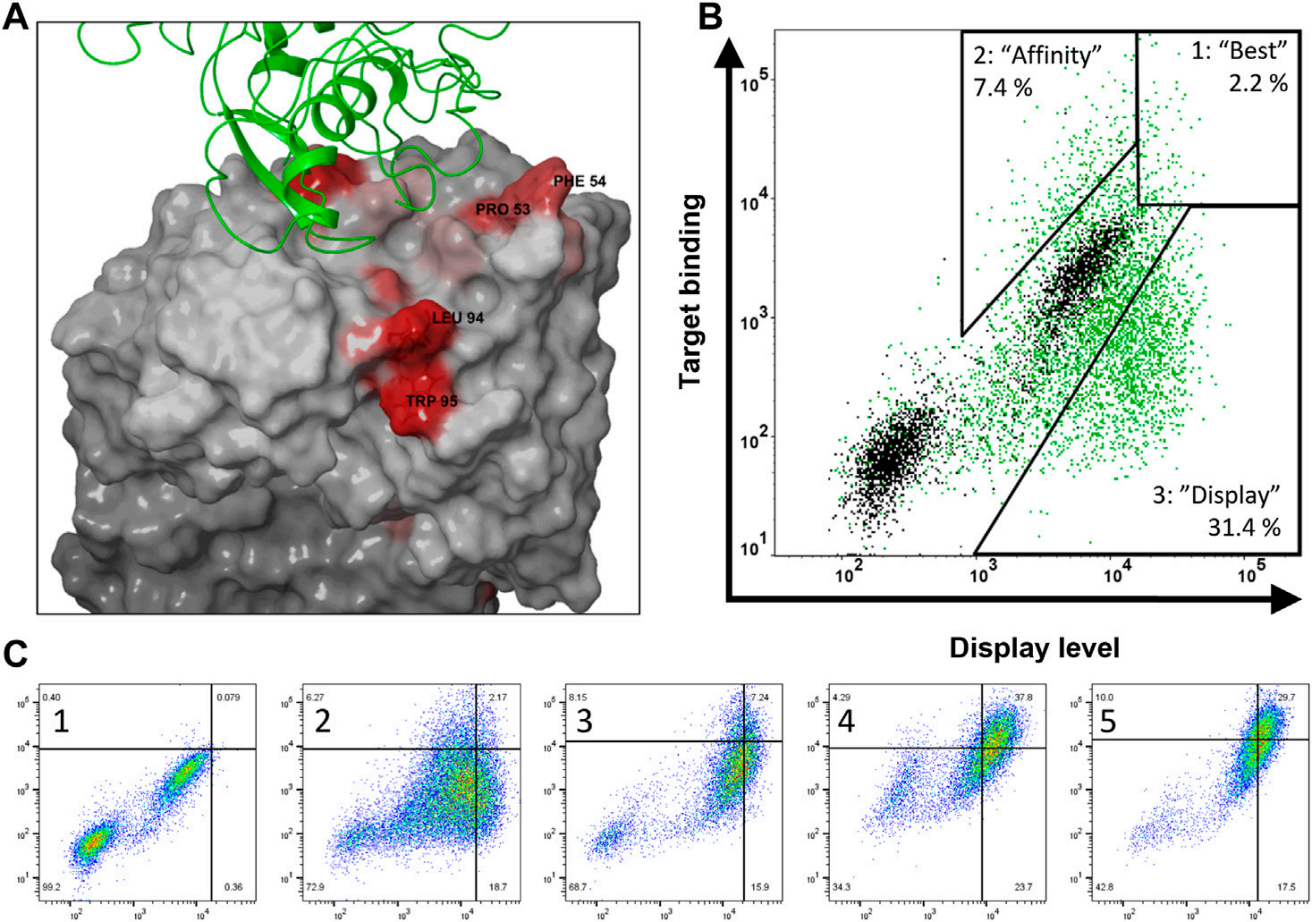
Structure-guided design and construction of bococizumab mutant library and selection of biophysically improved variants. **(A)** Crystal structure of bococizumab Fab bound to PCSK9 (PDB ID: 3SQO) with AggScore intensities shown in red. The two non-paratopic patches with the highest AggScore (PRO 53, PHE 54 in CDR-H2 and LEU 94, TRP 95 in CDR-L3) were randomized using NNK site-saturation mutagenesis. **(B)** Gating strategy of bococizumab mutant library FACS sorting to separate three distinct populations. Bococizumab is shown in black and mutant library first FACS sorting round is shown in green. Compared to bococizumab, gate 1 has both improved display levels and antigen binding levels, thus named “best”, gate 2 has improved antigen binding level only, thus named “affinity”, gate 3 has improved display level only, thus named “display”. **(C)** Enrichment of cells in the “best” gate through four rounds of FACS sorting (2–5). Gates were done using bococizumab displaying cells (1). Display level was measured using PE-anti IgG light chain antibody and target binding using mono-biotinylated PCSK9 and APC-conjugated monomeric streptavidin.

The crystal structure of bococizumab’s antigen binding fragment (Fab) bound to PCSK9 has been published (Liang et al., 2012). To identify problematic residues responsible for the bad biophysical properties of bococizumab, we probed the crystal structure of bococizumab’s Fab using Schrödinger BioLuminate platform to determine patches on the Fab surface with the highest AggScore. The AggScore predicts aggregation-prone regions using the distribution, orientation, and intensity of hydrophobic and electrostatic patches on the surface of the protein (Sankar et al., 2018). We identified two non-paratopic hydrophobic patches with high AggScore, one within the CDR3 of the variable light region (V_L_) (leucine 94; AggScore 15.0 and tryptophan 95; AggScore 14.3) and one within the CDR2 of the variable heavy region (V_H_) (proline 53; AggScore 7.9 and phenylalanine 54; AggScore 7.1). Importantly, based on the crystal structure, neither of these two amino acid patches are directly involved in the antigen binding and as such, mutating these residues should, in theory, have a low risk of negatively affecting the affinity towards PCSK9 (Figure 2A).

These two amino acid patches were targeted with degenerate NNK codons yielding a library with a theoretical diversity of 160,000 amino acid variants that was subsequently transfected into 2.4 × 10^8^ suspension adapted CHO-LP cells. Integration efficiency was determined by measuring the percentage of antibody expressing cells 8 days post transfection (dpt) from a sample of the transfected library growing in the absence of antibiotic selection. The measured integration efficiency was 1.7%, which meant that the number of transfected cells was 3.9-fold higher than the DNA diversity (see materials and methods) and 26-fold higher than the amino acid diversity of the constructed library. Thus, based on Poisson distribution, the expected coverage of the desired DNA diversity in CHO cells was 98%, and the probability of containing every possible desired amino acid variant was >99.99% (Patrick et al., 2003). Two weeks post transfection the library was subjected to magnetic-activated cell sorting (MACS) to enrich antibody expressing cells.

### Antibody display level was increased without loss of affinity

To investigate the correlation between antibody display levels, affinity and biophysical properties of antibodies displayed on mammalian cells, we used FACS to isolate three distinct populations from the bococizumab mutant library. Relative to parental antibody these populations showed: 1) increased display level and antigen binding level (named “best”), 2) increased antigen binding level only (named “affinity”), and 3) increased display level only (named “display”). Gating was made using the parental bococizumab as a control in every sorting round, as shown in Figure 2B. A subset of parental cells can be seen as non-expressing in Figure 2B, however, gating of library cells was not affected by this population. We hypothesized that if the antibody display level correlated with the biophysical properties and antigen binding level with affinity, we would isolate variants with the most overall favorable developability properties from the “best” gate, while the “affinity” gate would contain very high affinity variants with less favorable biophysical properties and variants with lower affinity but improved biophysical properties would be found in the “display” gate.

We stained 1 × 10^8^ MACS-enriched cells first with 10 nM mono-biotinylated PCSK9 and after washing excess antigen away, cells were stained with fluorescently labeled monomeric streptavidin to mitigate avidity effects due to high affinity of parental bococizumab. To ensure sufficient enrichment in each population, cell sorting was repeated twice with the “affinity” and “display” populations and four times with the “best” population, retaining similar gating strategies in each subsequent sorting round (Figure 2C). In the last sorting round of the “best” population, the antigen concentration was decreased to 1 nM and after a wash, a 100-fold excess of competing non-biotinylated PCSK9 was added with the fluorescently labeled streptavidin to add an off-rate selection step to select for the highest affinity clones.

### Next generation sequencing was used to monitor the enrichment of the variants

To analyze the quality of the library and to monitor the enrichment throughout the cell sorting process, genomic DNA was isolated from each round, antibody genes were amplified and subjected to next generation sequencing (NGS). In addition, samples were taken from the transfected library (pre-selection), and after MACS (MACS-sorted).

**FIGURE 3:**
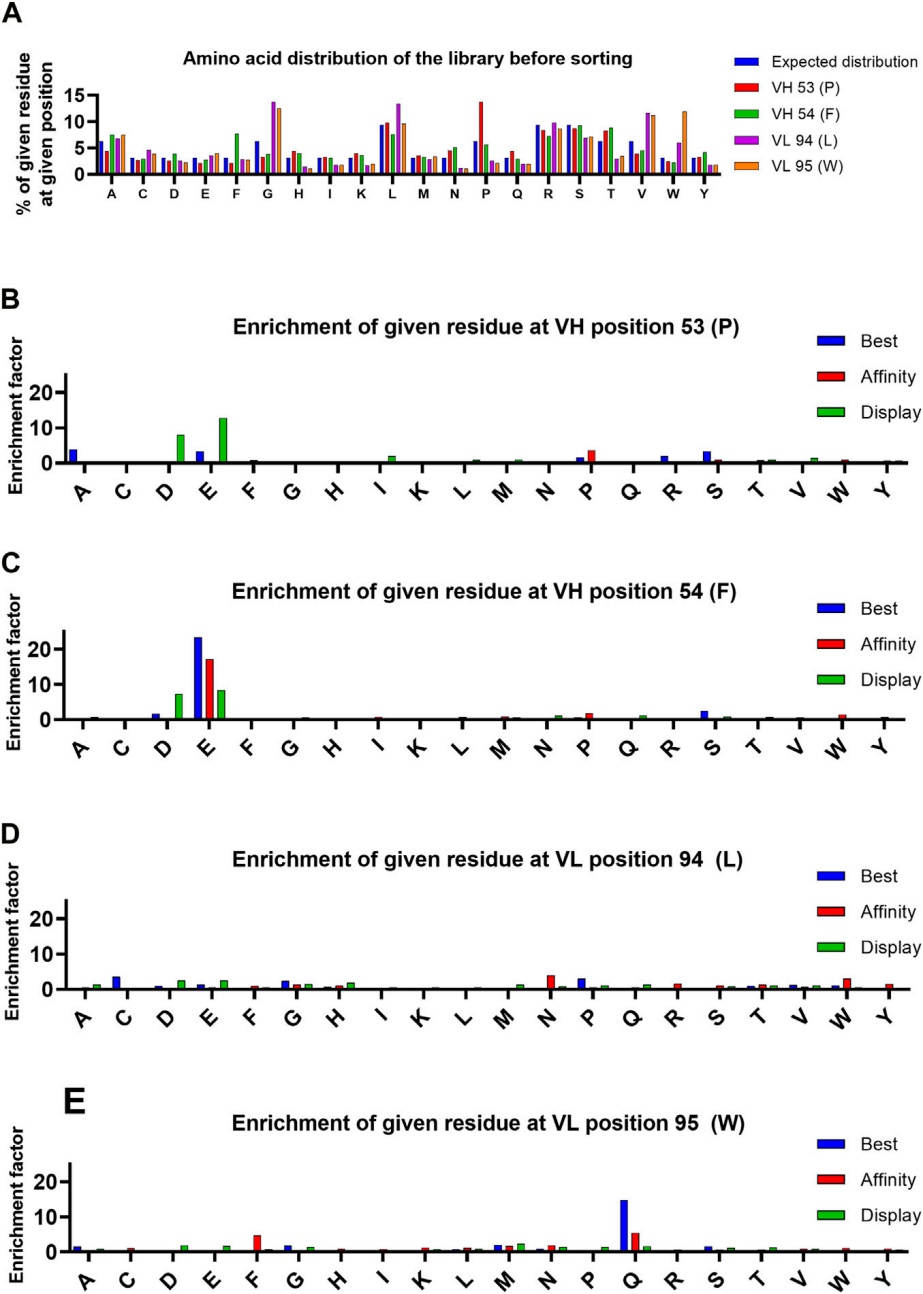
Analysis of amino acid distribution in the library before selection, and enrichment of amino acids at indicated positions during selection. **(A)** Distribution of amino acids in the library in each targeted position. Expected distribution is calculated from the possible codons of NNK mutagenized library. **(B–E)** Enrichment of each amino acid at the targeted positions. The enrichment is calculated by comparing the frequency of each amino acid in the pre-selection population and the final selection round of each population.

The amino acid distribution of the NNK randomized library prior to selection was close to the expected distribution except for the presence of slightly more parental bococizumab residues (Figure 3A). This might have been caused by slightly stronger hybridization of oligos within the degenerate oligo pool that matched exactly to the parental sequence resulting in more efficient amplification of the parental sequence during the site-saturation mutagenesis reaction. Despite this, the small excess of parental bococizumab sequence allowed us to observe the diminishment of parental sequence more clearly from the library population during the selection rounds; parental bococizumab sequence decreased during selection 16-fold from 1.3% to 0.08% using the “best” gating strategy (Table 1). As the gating barely excluded parental bococizumab, some bococizumab displaying cells remained in the “best” population, however, stricter gating likely would have excluded parental bococizumab entirely from the population.

**TABLE 1:** Enrichment factors and normalized count percentages of top variants in each of the three populations. Normalized count was determined by dividing the number of reads of the variant by the total number of reads in the sample. Enrichment factor was calculated by comparing the normalized count in the pre-selection sample and the final FACS sample. Variants in bold were selected for expression and characterization.

Population	V_H_ variant	V_L_ variant	Normalized count (%)	Enrichment factor
Best	**SE**	**GQ**	**18.46**	**11,700**
	**AE**	**GQ**	**2.53**	**6,800**
	**AE**	**PA**	**4.28**	**4,200**
	**PE**	**GQ**	**2.64**	**2,600**
	PE	VM	2.32	2,300
	PE	TS	1.47	2,300
	RS	CG	10.40	2,200
	PE	VS	5.13	1,500
Bococizumab	**PF**	**LW**	**0.08**	**0.06**
Affinity	**PE**	**RQ**	**4.33**	**2,700**
	**PE**	**NW**	**1.75**	**2,400**
	PE	VM	0.68	700
	PE	GC	0.88	500
	PE	SV	1.07	300
Display	**ED**	**GM**	**1.97**	**2,100**
	DD	HS	0.63	1,300
	DD	MG	0.40	500
	EQ	AT	0.44	200
	ED	GE	0.37	200

The bold values indicate variants that were expressed and characterized.

Enrichment of individual residues in each targeted position was analyzed for each of the three sorted populations (Figures 3B–E) to gain insight on the type of mutations that affected the display level and target binding. An enrichment factor was calculated for each targeted position by comparing the frequency of individual residues in the pre-selection population to the final FACS-sorted population. After the 4 sorting rounds, there were two individual mutations found in variants in the “best” gate population with an enrichment factor greater than 5: F54E in V_H_ (enrichment factor = 23) and W95Q in V_L_ (enrichment factor = 15). These same residues in these positions were the only mutations found to have conferred enrichment factors greater than 5 to variants in the “affinity” population after 2 sorting rounds, suggesting the target binding properties play a significant role in the enrichment of these residues in these positions. Analysis of variants found within the “display” gate led to identification of negatively charged residues (D and E) with enrichment factors greater than 5 in either targeted position of V_H_. Finding negatively charged residues enriched in the population with selection pressure only on display level is consistent with literature, which suggests that introducing aspartic acid or glutamic acid point mutations may improve the biophysical properties of an antibody (Dudgeon et al., 2012).

Conversely, two of the four targeted parental residues, F54 in V_H_ and L94 in V_L_, were eliminated by mammalian display, irrespective of gating strategy. The F54 in V_H_ and L94 in V_L_ were de-enriched 30-fold and 28-fold, respectively, in the “best” population during the 4 rounds of FACS strongly indicating that these residues were causing biophysical liabilities in bococizumab. Interestingly, while there was a strong preference to replace F54 with a negatively charged residue, L94 appeared to tolerate a variety of options.

**FIGURE 4:**
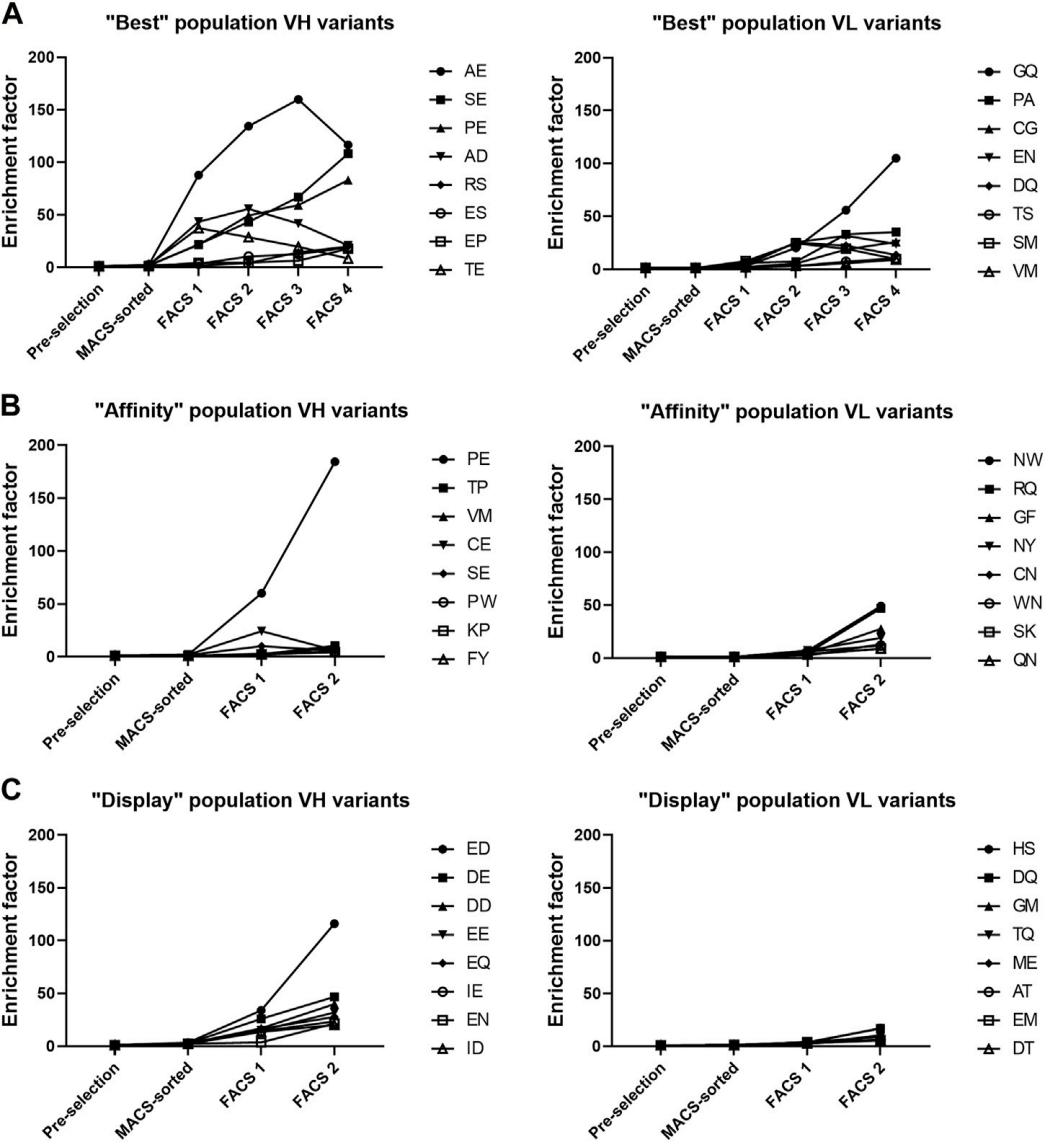
Top 8 most enriched V_H_ and V_L_ variants in each of the three populations. **(A–C)** Enrichment factor was calculated by comparing the frequency of each variant between the final FACS selection sample and the pre-selection sample.

Similar to analysis of single mutations, the enrichment of double amino acid mutations in either V_H_ or V_L_ regions allowed identification of combinations of mutations that led to enrichment of variants in the three populations. Enrichment factors throughout the selection process of the 8 most enriched V_H_ and V_L_ variants of each population are shown in Figure 4 and suggests that V_H_ variants were more enriched than the V_L_ variants. Three V_H_ variants stood out from the “best” population, each having enrichment factors >80, (AE, SE and PE named after the mutated residues in positions 53 and 54 of V_H_). The PE variant was the only variant also found greatly enriched in the “affinity” population, having an enrichment factor >180. The presence of the V_H_ variant PE in both the “affinity” and “best” population in combination with the absence of AE and SE variants in “affinity” population indicates that the P53 substitution with either alanine or serine retained the target binding while improving the display level and consequently the biophysical properties of the antibody, which was confirmed by biophysical characterization (Table 2). In the “display” population, V_H_ variants with negatively charged residues were the most enriched. Variant ED stood out from the rest, having the highest enrichment factor of 116.

**TABLE 2:** Summary of the biophysical characterization results of the selected bococizumab variants.

Category	Variant name	V_H_ CDR2	V_L_ CDR3	AC-SINS (nm)	Polyreactivity ELISA	PDI	HIC RT (min)	Tm (°C)	KD (pM)
Bococizumab	PFLW	EISPFGGRTNYNEKFKS	QQRYSLWRT	22.6	1.56	0.30	4.6	65.9	250
Best 1	SEGQ	...SE............	.....GQ..	1.1	0.77	0.04	3.9	67.0	350
Best 2	AEGQ	...AE............	.....GQ..	0.7	0.44	0.05	3.7	67.7	190
Best 3	AEPA	...AE............	.....PA..	0.8	0.43	0.05	3.7	67.4	210
Best 4	PEGQ	....E............	.....GQ..	1.4	0.53	0.19	3.7	68.5	200
Affinity 1	PENW	....E............	.....N...	1.2	0.49	0.07	3.7	67.2	240
Affinity 2	PERQ	....E............	.....RQ..	3.5	0.86	0.38	3.6	68.9	250
Display 1	EDGM	...ED............	.....GM..	0.2	0.15	0.14	3.8	67.5	660
Best V_L_ only	PFGQ	.................	.....GQ..	19.2	1.54	0.59	4.3	67.6	250
Best V_H_ only	SELW	...SE............	.........	2.5	0.87	0.05	4.1	65.8	270
F54 Point mutation	PELW	....E............	.........	1.4	0.72	0.46	3.9	66.6	190
Trastuzumab	-			0.5	0.15	0.03	4.4	70.6	-

The V_L_ variants were much less enriched and there were no clear trends seen (Figure 4). In the “best” population, variants GQ and PA were the most enriched, having enrichment factors of 105 and 35, respectively. In the “affinity” population, variants NW and RQ had enrichment factors of 49 and 46, respectively while in the “display” population there were no V_L_ variants with enrichment factors above 20. This suggests that the targeted V_H_ residues caused more biophysical liabilities than the targeted V_L_ residues.

Since the sequencing was done from a single amplicon containing the sequences of both targeted amino acid patches, we could pair the V_H_ and V_L_ variants to find the most enriched combinations of V_H_ and V_L_ mutations. From the “best” population, we picked 4 of the most enriched V_H_-V_L_ variants to be expressed and characterized (Table 1). We also picked 2 variants from the “affinity” population and one variant from the “display” population. We also expressed variants PFGQ, SELW and PELW to discern the effect of V_H_ and V_L_ mutations separately as well as the effect of F54E point mutation.

In addition to the enrichment factor, we normalized read counts for each variant by dividing the number of reads by the total number of reads in the sample. For example, the normalized read count of variant RSCG after the fourth FACS round in the “best” population was 10.4%, making it the second most frequent variant (Table 1). However, due to the biased NNK mutagenesis (see Figure 3A), the RSCG variant was present 13-fold more in the pre-selection population than the AEGQ variant, making the RSCG variant only the seventh most enriched variant, excluding it from the list of expressed variants. This example highlights the benefit of NGS when selecting antibodies from libraries with an uneven distribution of variants.

### Mammalian display was able to select antibodies with improved biophysical properties

To test if our mammalian display sorting campaign had selected antibodies with different biophysical properties based on display levels and antigen binding levels, we expressed and purified the parental bococizumab, along with the 10 selected variants. To determine whether the mutations had altered binding, we measured the affinities of the expressed bococizumab variants using surface plasmon resonance (SPR). As was expected, the affinities of the variants were almost identical to the parental bococizumab (Table 2). Only the EDGM variant, selected based on display level only, had slightly worse affinity due to a faster off-rate (Supplementary Figure S3). The measured affinity of bococizumab differed from the reported affinity (Liang et al., 2012), which was determined using a different technology and different methodology with PCSK9 as the immobilized ligand rather than soluble analyte. However, by comparing the affinity measurements of the expressed variants against that of bococizumab, we could show that the affinities of the variants were not changed significantly.

The most problematic biophysical properties of bococizumab were its self-interaction propensity and polyreactivity (Jain et al., 2017). To test whether the selected variants had better biophysical properties compared to the parental bococizumab, we subjected the variants to several biophysical assays including affinity-capture self-interaction nanoparticle spectroscopy (AC-SINS), polyreactivity ELISA, analytical hydrophobic interaction chromatography (HIC) and nano differential scanning fluorimetry (nanoDSF) with dynamic light scattering (DLS).

AC-SINS is an assay measuring the colloidal stability of antibody-coated gold nanoparticles (Wu et al., 2015). The colloidal stability is dependent on the propensity of the analyzed antibody to self-interact, which can be monitored by measuring the plasmon wavelength of the particles. The plasmon wavelength is red-shifted with antibodies with high attractive self-interaction propensity compared to particles without analyte antibody. In our measurements the AC-SINS score of bococizumab was 22.6 while the mammalian display-selected variants showed greatly improved scores (Table 2). Variant EDGM, which was selected from the “display” population had the best AC-SINS score of 0.2, even better than that of Trastuzumab which was used as a control due to its good biophysical characteristics (Jain et al., 2017).

**FIGURE 5:**
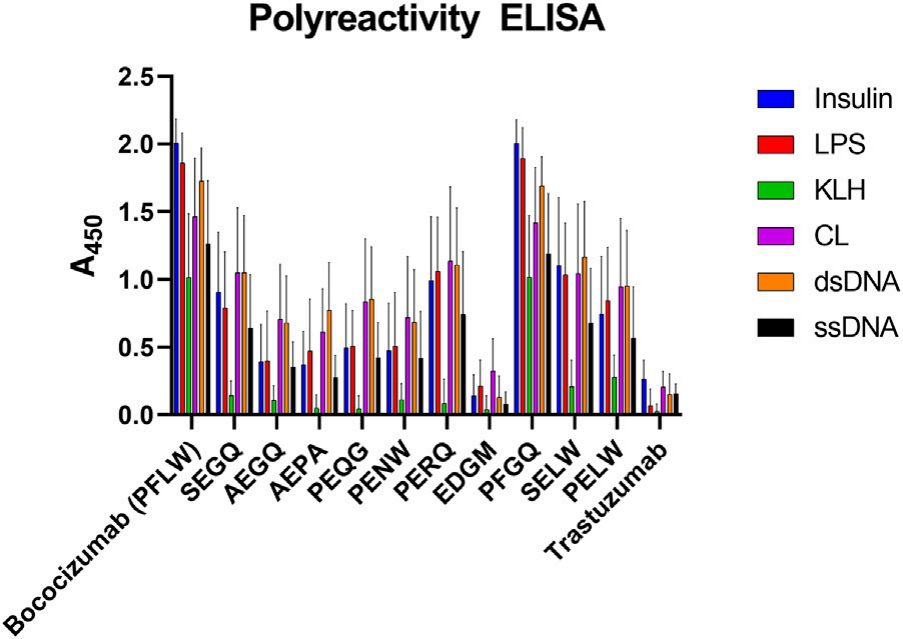
Polyreactivity ELISA results of bococizumab variants by antigen. The scores are averages from four samples of a dilution series and the error bars depict the standard error of the mean. LPS, lipopolysaccharide; KLH, keyhole limpet haemocyanin; CL, cardiolipin; dsDNA, double-stranded DNA; ssDNA, single-stranded DNA.

The polyreactivity ELISA highlighted bococizumab’s propensity to bind non-target proteins (Figure 5). The EDGM variant selected for display levels again performed the best, scoring the same as Trastuzumab while the remaining mammalian display-selected variants also exhibited significantly reduced polyreactivity (Table 2). The polyreactivity ELISA scores had a very significant correlation with the AC-SINS scores (Spearman r = 0.94, *p* < 0.0001), which is in line with a previous study by Dobson *et al.*, where they showed that mutating individual amino acids of an antibody can simultaneously reduce aggregation and polyreactivity while increasing the *in vivo* half-life (Dobson et al., 2016).

We confirmed the aggregation propensity of bococizumab using DLS. DLS measures the size distribution profile of the sample with the most useful parameter from the DLS measurement in this context being the polydispersity index (PDI) describing the dispersity of the sample. The smaller the PDI value, the more monodisperse i.e., less agglomeration or aggregation. We first analyzed the expressed antibodies immediately after the protein A and size exclusion chromatography (SEC) purification, but as the possible aggregates were removed, there were no differences in the PDI values of the samples (data not shown). As SEC purification is usually omitted from high-throughput screening of antibodies, we wanted to test whether we could see differences in the aggregation propensity in samples where the aggregates were not removed by SEC. To this end we made another small-scale expression batch of the same antibodies, but only purified them using protein A spin columns. This time we could see clear differences between the PDI values of the variants (Table 2). The variants from the “best” population had the lowest PDI scores. There was a significant correlation between the PDI values and the AC-SINS scores (Spearman r = 0.67, *p* = 0.02).

To discern the effect of the mutations on the thermal stability, we determined the melting temperatures (T_m_) of the bococizumab variants using nanoDSF. The T_m_ of the mammalian display-selected variants were increased 1.1°C–3.0°C compared to bococizumab. Based on the results, the V_L_ mutations played a bigger role on the thermal stability of the variants than the V_H_ mutations. For example, when V_H_ residues were mutated from PF to SE without reciprocal V_L_ mutations, the T_m_ was decreased by 0.1°, but when the V_H_ with SE was combined with the V_L_ having mutations GQ, the Tm was increased by 1.2°. Similarly, when V_L_ residues were mutated from LW to GQ in the absence of any V_H_ mutations, the T_m_ was increased by 1.7° (Table 2). The more prominent role of the V_L_ mutations on antibody stability could be related to their proximity to the V_L_-V_H_ interface, whereas the V_H_ mutations resided in the tip of the CDR-H2 away from the interface between chains.

The patches in bococizumab with the highest AggScore were hydrophobic. We analyzed bococizumab and the selected variants with analytical hydrophobic interaction chromatography (HIC) to experimentally evaluate variants for differences in hydrophobicity. All mammalian display-selected bococizumab variants had a decreased HIC retention time (RT), indicating reduced hydrophobicity. Interestingly, both V_H_ and V_L_ mutations contributed to the decrease in hydrophobicity as designed by the library.

The targeted residues L94 and W95 in the V_L_ had very high AggScores whereas the P53 and F54 in the V_H_ had only half of that. Interestingly, the mutated V_H_ residues, especially the F54, had the most impact in the AC-SINS, polyreactivity ELISA and PDI scores, which are all assays related to the measurement of aggregation and non-specific binding. While mutation of V_L_ residues had almost no impact on polyreactivity and self-interaction (Table 2) V_L_ mutations did have a much bigger impact on the T_m_ and could be connected to the observed improvement in HIC. Thus, while most biophysical liabilities could be fixed with a single F54E point mutation, additional mutations appeared to have a cumulative beneficial effect on the biophysical properties of bococizumab.

### *In silico* assessment of the improved bococizumab variants

**FIGURE 6:**
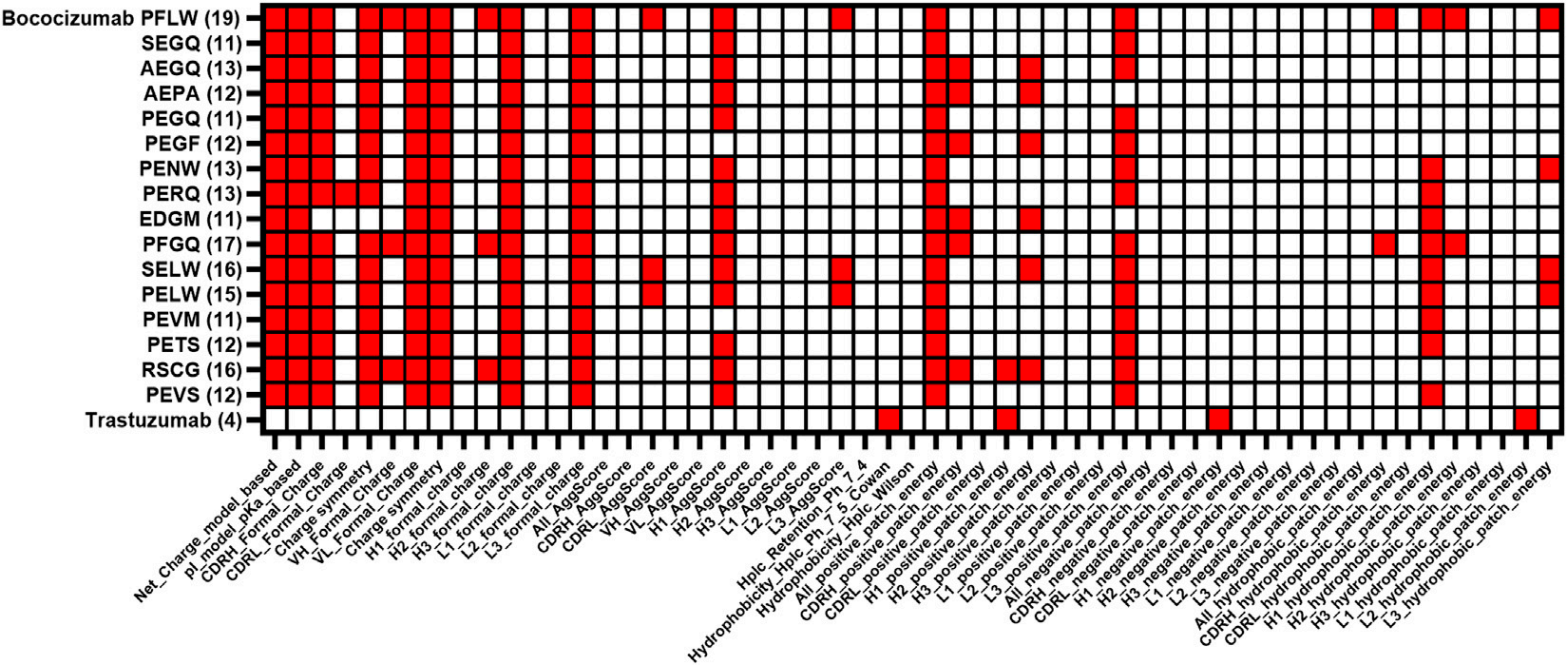
Comparison of protein descriptor liability flags of characterized bococizumab variants together with the most enriched clones from the “best”. The protein descriptor value was flagged if the value was higher than the average + standard deviation of the value of the 48 approved therapeutic antibodies from Jain et al. (Jain et al., 2017). The total number of flags for each variant is in brackets.

After characterization of the selected bococizumab variants, we performed a retrospective *in silico* analysis of the characterized variants and the 4 most enriched variants from the “best” population that did not make it to the expression and characterization phase (Table 1). We wanted to see how well the computational modelling could predict the biophysical properties of the selected antibody variants using only amino acid sequence as input. The retrospective *in silico* analysis was done using the antibody structure prediction and protein descriptor calculations with Schrödinger BioLuminate using the variable domain sequences as input. Out of the over 900 protein descriptors calculated by BioLuminate, we chose 55 of the most relevant parameters, which were related to the antibody’s charge, isoelectric point, AggScore, electrostatic patch energy and hydrophobic patch energy for each CDR separately. We did similar structure prediction and protein descriptor calculations for the 48 approved antibodies from the study by Jain et al. (Jain et al., 2017) and calculated their average values to determine cut-offs for each parameter, the cut-off for each parameter was defined as the average score plus standard deviation of the modelled approved therapeutic antibodies. This way we were able to flag potentially problematic parameters for each of the analyzed bococizumab variants (Figure 6). The number of flags was reduced from 19 for bococizumab to 11–13 for all the mammalian display-selected variants. In comparison, the average number of flags for the 48 FDA approved antibodies was 8. The RSCG variant, which was covered by over 10% of the reads in the “best” population after 4 rounds of FACS, but which was less enriched than the other variants, had 16 flags. This suggests that the selection of variants based on the enrichment rather than normalized counts was the better option in an NNK randomized library with strong bias towards amino acids encoded by more codons.

To evaluate the reliability of the protein descriptor calculation, we gave an overall ranking score to the variants by ranking them on the individual biophysical assays and summing up the individual ranks. The number of flags had a significant correlation with the overall ranking scores (Wilcoxon *p* = 0.0005), suggesting that the number of flagged protein descriptors could be a useful parameter for ranking a large number of hit antibodies using only sequence as input. This approach could be utilized when choosing which antibodies or variants to pursue after mammalian or e.g., phage display selection. The antibody structure prediction and protein descriptor calculations can be performed easily for dozens to hundreds of variable domains within a day using Schrödinger BioLuminate on a normal laptop.

## Discussion

Mammalian display is a useful tool in antibody engineering due to its inherent protein production quality control machinery that can filter out antibodies with biophysical liabilities. In this study, we show a straightforward method for generating a CHO cell line suitable for mammalian display and demonstrate its capability of selecting antibodies with favorable biophysical properties based on the antibody display level. We corroborated the antibody enrichment analysis with thorough biophysical characterization of the mammalian display-selected antibodies that confirmed the filtering ability of mammalian display technology.

The Flp-In CHO line has several attractive features and has been used extensively for stable transgene expression thereby negating the need for a thorough cell line stability study. It has also been used previously for mammalian display (Zhou et al., 2010), however, as shown here, multiple copies of LP can be integrated (Figure 1) thereby potentially leading to conflation of variant properties during selection. When combined with low efficiency of stable transfections of the Flp-In system (Yamaguchi et al., 2011; Parthiban et al., 2019), it was clear that additional engineering was required. Here, we increased fidelity and efficiency of the system by integrating Bxb1 LP into the parental Flp-In CHO line leading to increased efficiency while ensuring a single copy of each variant is contained per cell.

The biggest limitation of mammalian display compared to other display technologies is the limited size of the displayed library. This is simply because the mammalian cells are much bigger than other systems such as yeast and phage display. As CHO cells are routinely cultured in 0.2–10 × 10^6^ cells/mL density, maintaining a mammalian display library of 10^9^ diversity would require a culture volume of several liters. For example, using our current transfection protocol which yielded a 1.7% stable integration efficiency, we would need 150-L of cell culture to have a high probability of containing 10^9^ library diversity. To this end, we have made initial experiments for replacing the lipofection with electroporation and got 4-fold improvement in integration efficiency using a cuvette based Nucleofector (data not shown). Others have further increased their transfection efficiency by switching from Nucleofector to MaxCyte electroporation (Parthiban et al., 2019), which also enables the transfection of up to 20 × 10^9^ cells at a time using flow electroporation. With these improvements, we expect our mammalian display to be capable of displaying libraries with diversities of tens of millions antibody variants. It should also be noted that by using either codon-based or fully synthetic libraries, each amino acid will be encoded by a single codon, which decreases the DNA diversity of a library and removes the bias introduced by degenerate oligonucleotides.

Another strategy that circumvents culture size limitations would be to use phage display using large primary libraries to reduce diversity followed by subsequent conversion to IgG display format for mammalian display as has been described previously (Parthiban et al., 2019). The design of our targeting vector allows for a similar strategy whereby conversion of V_L_-V_H_ format scFvs from phage display into full-length IgG in mammalian display format can be performed by DNA assembly reactions (Gibson, HiFi etc.), while maintaining chain pairing, if desired.

The biophysical properties of bococizumab have been improved using a random mutagenesis strategy in combination with HEK293-based mammalian display by Dyson et al. (Dyson et al., 2020). Interestingly, in part of their study V_H_ (F54) was targeted for diversity with similar enrichment of negatively charged amino acids D and E. They also reported additional biophysical benefits from V_L_ mutations but without thorough analysis of combinatorial mutations as seen here. Importantly, despite their mammalian display system using HEK293 cells, results were similar. Thus, the selection of biophysically favorable antibodies based on the display level is not limited to HEK293 cells and could possibly be utilized in other mammalian cell lines, if desired.

Another recent case study where the biophysical properties of bococizumab were successfully improved used yeast display and machine learning (Makowski et al., 2022). In this study the selection for antigen binding and low self-association was performed separately using FACS and both positive and negative sequences from both selections were used to train a machine learning model. The model was then successfully used to identify rare co-optimal bococizumab variants with both retained affinity to PCSK9 and low self-association propensity. Similarly to earlier studies and in the present study, negatively charged residues were enriched in the CDRH2 of the optimized bococizumab variants. While this study is an impressive example of using machine learning in isolating rare co-optimal sequences from a yeast display library, one could argue that similar results can be achieved even without the use of machine learning, as demonstrated in the present study, by using mammalian display. It is hypothesized that self-interacting antibodies displayed at high density on mammalian cell surface are removed by the quality control machinery of mammalian cells (Apaja and Lukacs, 2014; Sun and Brodsky, 2019; Dyson et al., 2020), thus negating the need for additional self-association selection.

We wanted to gain insight on the type of variants mammalian display favors and in which way. In addition to finding antibodies with high affinity and good biophysical properties by having the selection pressure on both display level and antigen binding, we hypothesized that the platform could be used to find variants with either excellent affinity or excellent biophysical properties using a gating strategy that prioritized only antigen binding or display level. Indeed, the EDGM variant from the “display” population (that lacked selective pressure on affinity) showed the greatest mitigation of self-interaction and polyreactivity, while having only slightly reduced affinity compared to the parental bococizumab. Sometimes very high affinity can lead to restricted localization and tumor penetration (Adams et al., 2001) or reduced effector functions (Mazor et al., 2016), in which case biophysical optimization with selection pressure focused mostly on the display level could be useful. Furthermore, a recent publication demonstrating that reducing affinity actually increased immunomodulatory antibody agonism points to the utility of gating for specific needs as is possible here (Yu et al., 2023).

We were not able to find antibody variants with significantly improved affinity that exacerbated the already poor biophysical properties of bococizumab even with selective pressure put only on affinity. This was not surprising since library design was structure-guided to ensure none of the very limited number of residues targeted for mutagenesis were involved in direct interactions with the antigen (Figure 2A). Moreover, bococizumab itself already has very high affinity, which potentially limits the number of mutation options contributing positively to the affinity. To investigate the approach of finding extremely high affinity binders with sub-optimal biophysical properties using mammalian display, an expanded library design better suited for affinity maturation should be used.

We performed a retrospective *in silico* analysis of the most enriched antibody variants to evaluate the usefulness of ranking antibody variants based on modelling using the variable domain sequence as input. Setting thresholds using approved therapeutic antibodies, we were able to achieve strong correlation between the number of flagged protein descriptors and overall ranking of characterized variants. This suggests that the protein descriptor-based ranking could be useful for narrowing down the number of potential antibodies identified during selections that would need further characterization. The reliability of the modelling, however, is case-dependent but based on our limited experience, the protein descriptor ranking successfully highlighted possible biophysical liabilities in our antibody variants.

The sequence linked biophysical property data obtained from mammalian display could potentially be used for training deep learning based tools for antibody optimization. As demonstrated by Mason et al., a data set obtained from a relatively small mammalian display library could allow the design of deep learning-based virtual libraries for finding novel useful variants (Mason et al., 2021). The gating strategy shown in our study could possibly be used for gathering data from antibodies with different affinity and biophysical properties for training a deep learning algorithm for virtual screening of antibody variants with both high affinity and optimal biophysical properties. This would, however, require a different library construction strategy to obtain information about the relationship between the biophysical properties and a broader range of mutations.

Lastly, the mammalian display maturation campaign described in this study appears to have successfully improved the biophysical properties of bococizumab. If this were a campaign to develop a therapeutic antibody, this extensive biophysical characterization of select variants would be subjected to testing of *in vitro* biological activity suitable for the target as described previously (Liang et al., 2012). This would be followed by *in vivo* testing together with safety studies to identify potential target organs sensitive to toxicity as well as to guide safe dosing for clinical trials. It should be noted that in maturation campaigns, many resources are spent rank ordering variants for biophysical suitability in order to choose appropriate candidates and avoid costly and time-consuming *in vivo* studies. As described here, mammalian display can reduce this burden by acting as a filter for poorly behaved variants thereby speeding up the process of drug development.

In summary, the corroboration of rational design of an antibody optimization library using *in silico* tools and variant selection using mammalian display was shown to be successful for improving the biophysical properties of a failed therapeutic antibody. While the *in silico* tools are good for pointing out problematic residues and possible single point mutation solutions, they presently lack accuracy to model combinatorial mutations. As mammalian display allows the screening of millions of antibody variants to select variants with good biophysical properties, the identification of problematic patches and subsequent rational design of an optimization library is a useful tool in the therapeutic antibody engineering toolbox.

## Materials and methods

### Cell culture

Adherent Flp-In CHO cell line (Invitrogen, RRID:CVCL_U424) was cultured in Ham’s F-12 Nutrient Mix (Gibco) supplemented with 2 mM GlutaMAX (Gibco) and 10% fetal bovine serum (Sigma-Aldrich) at +37°C, 5% CO_2_ according to the manufacturer’s recommendations. The serum-free suspension adapted landing pad cell line was cultured in FectoCHO CD Medium (Polyplus), supplemented with 2 mM GlutaMAX at +37°C, 5% CO_2_ at 120 rpm orbital shaker with 19 mm throw. ExpiCHO cells (Gibco, RRID:CVCL_5J31) were cultured in ExpiCHO Expression Medium (Gibco) at +37°C, 8% CO_2_.

### Vector construction

Bxb1 landing pad (LP) vector was constructed to facilitate the integration of an antibody display cassette to the genome of the host cell using Bxb1 integrase for recombinase mediated cassette exchange (RMCE). In the LP construct, CMV promoter drove the expression of mouse IgG2a Fc surface marker followed by platelet-derived growth factor receptor *ß* (PDGFRβ) transmembrane (TM) domain that anchors it to the cell membrane. Blasticidin S deaminase (BSD) encoding gene was placed downstream of the surface marker, separated by furin cleavage site and P2A peptide, to enable the antibiotic selection of successful transfectants. The expression cassette was flanked by Bxb1 AttP and AttPm recombination sites for RMCE of the expression cassette (Figure 1). The landing pad vector was synthesized by GeneArt (Thermo Fisher) with flanking restriction sites for NheI and SphI, which allowed the restriction cloning to pcDNA5/FRT vector (Invitrogen) to create pcDNA5/FRT-LP vector.

To facilitate Bxb1 integrase driven RMCE, we constructed a promoterless Bxb1 targeting vector, pBxb1-TV-GFP, with AttB and AttBm recombination sites flanking the expression cassette. The pBxb1-TV-GFP was a bicistronic vector, which was used for initial testing and platform optimization, and consisted of DNA encoding human insulin signal peptide, EGFP with human CD8 stalk and CD8 TM domain fusion to anchor the protein to the cell membrane. Gene encoding puromycin N-acetyltransferase (PuroR) was placed downstream of the TM domain, separated by furin cleavage site and P2A peptide, to enable drug selection of stable integrants. Bovine growth hormone (bGH) polyA was placed downstream of the PuroR gene. Bococizumab expression cassette consisting of DNA encoding bococizumab light chain, furin cleavage site, T2A peptide, IGHV3 signal peptide and bococizumab heavy chain was cloned to the pBxb1-TV-GFP by replacing the EGFP encoding gene by NotI/HindIII (NEB) digestion and ligation to construct pBxb1-TV-Bococizumab. The Bxb1 integrase expression vector, pBxb1-EV, consisted of DNA encoding CMV promoter, SV40 NLS, HA tag, Bxb1 integrase and bGH-polyA. All the above expression cassettes were synthesized by GeneArt. Genes encoding membrane anchored BFP1.2 and dsRed were synthesized and cloned into pBxb1-TV by Twist Biosciences.

### Generation of suspension adapted landing pad cell line

To generate a suitable CHO cell line for mammalian display, the Bxb1 landing pad was integrated to the genome of the host cell line. Flp-In CHO cells were co-transfected with pcDNA5/FRT-LP and pOG44 (Invitrogen) at a ratio of 1:9 using Lipofectamine 2000 (Invitrogen) according to the manufacturer’s protocol. Two days post transfection (dpt), 600 μg/mL Hygromycin B (Gibco) was added. After the cells reached confluency, the cells were detached with Cellstripper (Corning), resuspended in PBS with 2% fetal bovine serum and stained with PE-Anti-mouse IgG2a Fc antibody (Jackson ImmunoResearch Labs Cat# 115-117-186, RRID:AB_2632510) according to manufacturer’s recommendations. The cells were cloned by single cell sorting to 96-well plates with 67 µL fresh media and 33 µL conditioned media, using BD FACSMelody (BD Biosciences). The sorting gate was made to select cells with the highest PE signal. Seven days after the sorting, 12 clones were expanded to 6-well plates and a master plate of the clones was frozen. The plated clones were co-transfected with 1:1 ratio of pBxb1-EV and pBxb1-TV-GFP. Puromycin selection (10 μg/mL) was added 2 dpt and after 14 days, the cells were stained with PE-Anti-mouse IgG2a Fc antibody as before and analyzed for mEGFP and mouse IgG2a Fc expression using BD Accuri C6 Plus (BD Biosciences). One of the clones which had high mEGFP signal, and no PE signal was selected as the landing pad cell line (CHO-LP). To verify the fidelity of the Bxb1 RMCE system, the CHO-LP cells were co-transfected with a mixture of two or three different membrane anchored fluorescent protein genes (BFP1.2, EGFP and dsRed) each encoded in pBxb1-TV, along with pBxb1-EV. After 8 days under drug selection, the cells were imaged with Nikon Ti2 fluorescence microscope using three fluorescence channels and brightfield channel. All channels were merged to form an image showing the expression of all fluorescent proteins in one image. After 10 days under drug selection, the cells were analyzed on NovoCyte Quanteon flow cytometer (Agilent) to determine the percentage of positive cells for each fluorescent protein. The flow cytometry data was analyzed using FlowJo software (BD Biosciences).

The serum-free suspension adaptation was performed by detaching the CHO-LP cells with Trypsin-EDTA 0.05% (Gibco) and seeding the cells to 0.5 × 10^6^ cells/mL to ProCHO4 protein-free CHO medium (Lonza) supplemented with 2 mM GlutaMAX and 0.2% anti-clumping agent (Gibco) at +37°C, 5% CO_2_ and shaken at 120 rpm. The cells were passaged to 0.5 × 10^6^ viable cells/mL every 3–4 days until the viability was >90% for 3 consecutive passages. The cells were subsequently adapted to FectoCHO CD Medium (Polyplus) for better transfectability.

### Bococizumab library design and construction

The Protein Data Bank file of bococizumab bound to PCSK9, (PDB ID: 3SQO) (Liang et al., 2012) was prepared with Protein Preparation Wizard in BioLuminate (Schrödinger Release 2022-3: BioLuminate, Schrödinger, LLC, New York, NY, 2022). The Protein Surface Analyzer tool was used to identify surface patches with the highest AggScore. A single bococizumab mutant library was constructed where L94 and W95 codons in the variable light region (V_L_) and P53 and F54 codons in the variable heavy region (V_H_) were randomly mutated using NNK oligonucleotide directed mutagenesis. A 694 bp PCR product containing the mutated patches in V_L_ and V_H_ and the sequence between (rest of light chain, furin cleavage site, T2A, IGHV3 signal peptide, beginning of V_H_), was amplified using the pBxb1-TV-Bococizumab as template using primers: forward: 5′-GCCAGCAAAGATATT CTNNKNNKCGGACATTCGGGCAAGGA-3′ and reverse: 5′-TTGGTCCGTCCGCCMNNMNNGGAGATTTCGCCCATC CA CTC-3’. The rest of the vector was amplified with primers: forward: 5′-GGC​GGA​CGG​ACC​AAC​TAC​A-3′, and reverse: 5′-AGA​ATA​TCT​TTG​CTG​GCA​ATA​GTA​TGT​TGC-3′ to have 17 bp and 14 bp overlaps upstream and downstream of the mutagenized PCR product, respectively. The mutagenized PCR product was inserted to the amplified vector backbone using NEBuilder^®^ HiFi DNA Assembly Master Mix (NEB) which was subsequently electroporated into NEB^®^ 5-alpha Electrocompetent *E. coli* (NEB). Dilutions of the transformation mixture were plated to ampicillin agar plates and individual ampicillin-resistant colonies were counted to confirm that the library size was more than 10-fold larger than the theoretical DNA diversity of the constructed library (1.05 × 10^6^). The rest of the transformation mixture was plated on ampicillin agar plates, from where the colonies were scraped into 50 mL LB-ampicillin the next day. Transfection-quality plasmid DNA was prepared with Plasmid Plus Midi Kit (Qiagen) from the liquid culture after 3 h incubation at +37°C, 250 rpm.

### Library transfection, cell staining and sorting

The day before transfection, 120 × 10^6^ suspension adapted CHO-LP cells were centrifuged for 5 min at 250 x *g* and resuspended to 1 × 10^6^ cells/mL in fresh medium and cultured in a 500 mL Erlenmeyer shake flask (Corning) in 120 rpm orbital shaker with 19 mm throw. On the day of transfection, 48 µg of the constructed bococizumab library was co-transfected with 12 µg of pBxb1-EV into the cells using FectoPRO^®^ transfection kit (Polyplus) according to manufacturer’s instructions. Puromycin selection (5 μg/mL) was added 2 dpt to select stably transfected cells. The puromycin concentration was increased to 10 μg/mL at 5 dpt and the cells were maintained at a viable cell density of 0.2-6 x 10^6^ cells/mL with media changes every 3–4 days without cell passage to maintain full diversity. During the selection, viability dropped to <15% at which point dead cells were removed using slow centrifugation at 50 *g* for 10 min to pellet live cells while leaving dead cells in suspension. Stable transfection efficiency was measured 8 dpt from a sample of the transfected library cultured without drug selection by staining the cells with PE anti-human Ig light chain κ (BioLegend Cat# 316508, RRID:AB_493613) and measuring the percentage of antibody displaying cells using BD Accuri C6 Plus flow cytometer. Two weeks after transfection, antibody expressing cells were MACS enriched by staining with PE anti-human Ig light chain κ and using anti-PE MicroBeads (Miltenyi Biotec), according to the manufacturer’s instructions. The cell staining was done in EasySep buffer (Stemcell technologies) at 50 × 10^6^ cells/mL on ice for 30 min for each staining with washing between, according to manufacturer’s recommendations. When performing staining also with the target protein, the first staining was done using 10 nM mono-biotinylated PCSK9 (AcroBiosystems) and the second staining using PE anti-human Ig light chain κ and SAvPhire (monomeric streptavidin, Sigma-Aldrich) which was conjugated using APC Conjugation Kit—Lightning-Link (Abcam), according to the manufacturer’s recommendations. When performing off-rate selection, 1 µM non-biotinylated PCSK9 (AcroBiosystems) was added to the second 30-min staining reaction. The stained cells were resuspended to 5 × 10^6^ cells/mL in EasySep buffer and sorted at 500–2000 cells/sec into 5 mL tubes with 1 mL medium using BD FACSMelody Cell Sorter, where cells were sorted based on display level using PE signal and antigen binding using APC signal. The gates for sorting were done using cells displaying bococizumab, as shown in Figure 2. Between 1 × 10^5^ and 3 × 10^6^ cells were collected for the populations each round and the cells were transferred to 6-well plates to recover. After each round of sorting, the cells were let to grow for 12–17 days, and 5 × 10^6^ cells were pelleted and frozen for DNA extraction before the next sorting round.

### Recovery of antibody genes and next generation sequencing

Genomic DNA was extracted from the frozen cell pellets using Monarch^®^ Genomic DNA Purification Kit (NEB). To enable the pairing of the mutated regions in V_L_ and V_H_, an amplicon consisting of only the mutated regions and the sequence between them was amplified as a single amplicon. The 778 bp amplicon was amplified using oligonucleotides which hybridized directly upstream of the mutagenized patch in V_L_ and directly downstream of the mutagenized patch in V_H_. The oligonucleotides contained universal adapter sequences in the 5′ end, which allowed the insertion of index adapters by PCR using Nextera Index kit (Illumina) (forward: 5′- ACA​CTC​TTT​CCC​TAC​ACG​ACG​CTC​TTC​CGA​TCT​GCA​ACA​TAC​TAT​TGC​CAG​CAA​AGA​TAT​TC -3′, reverse: 5′- GAC​TGG​AGT​TCA​GAC​GTG​TGC​TCT​TCC​GAT​CTT​TGT​AGT​TGG​TCC​GTC​CGC​C -3′). To ensure the coverage of the full diversity, 15, 3 or 1 × 50 µL PCR reactions with 1,000 ng template in each reaction were prepared for the pre-selection, MACS-sorted and the rest of the samples, respectively, using NEBNext Ultra II Q5 Master Mix (NEB). The amplicons were sent to Eurofins Genomics where the index adapters were added and library was prepared and sequenced on Illumina MiSeq platform using the MiSeq Reagent Kit v3 (600 cycles, paired-end). The sequences were annotated, and the variants were clustered with 100% identity in the mutated areas using PipeBio online platform. After annotation and clustering, there were 630,000—700,000 correctly annotated V_H_ or V_L_ reads for pre-selection and MACS-sorted samples and 18,000—255,000 V_H_ or V_L_ reads for the FACS-sorted samples. The normalized count was calculated for each cluster in each sample by dividing the number of reads in the cluster by the total number of reads in the sample. The enrichment was calculated after each sorting round by dividing the normalized count of the cluster with the normalized count of that cluster in the pre-selection sample. The light chain (LC) and heavy chain (HC) genes of the selected variants were synthesized separately and cloned into pTwist CMV vector by Twist Biosciences.

### Antibody production and purification

The bococizumab variants were expressed transiently in ExpiCHO cells using ExpiFectamine kit (Thermo Scientific) following the manufacturer’s high yield protocol, using 1:1 ratio of LC to HC plasmid. The expression was done in 10 mL scale using TubeSpin Bioreactors 50 (TPP) in 200 rpm orbital shaker with 19 mm throw using 45° angle. The product was harvested 11 dpt by two consecutive centrifugations (300 x *g*, 5 min, 20°C) leaving the cell pellet in the tube before the second centrifugation (11,000 x *g*, 20 min, 4°C), followed by filtration through a 0.22 µm filter. The antibodies were purified in a sequential affinity and size-exclusion chromatography procedure using Äkta Pure (Cytiva). The cell culture supernatant was first loaded to a HiTrap MabSelect PrismA column (Cytiva), eluted to a 2 mL loop with 0.1 M sodium citrate pH 3.0 and loaded to a Superdex200 Increase 10/300 GL column (Cytiva) with PBS. Fractions were collected to 96-well deep well plates, from where the antibody containing fractions were pooled. Another batch of the bococizumab variants was produced for the PDI and HIC measurements similarly but using a 2:1 ratio of LC to HC plasmid and purifying the proteins using only NAb™ Protein A Plus Spin Columns (Thermo Fisher) and buffer exchanging the antibodies to PBS after purification using 40 kDa MWCO Zeba spin desalting columns (Thermo Scientific). Commercial Trastuzumab (Roche) was used as a control in the biophysical assays.

### Affinity measurements with surface plasmon resonance

Kinetic analysis of the antibody variants was performed with surface plasmon resonance using Biacore 8K + instrument (Cytiva). A capture assay was used to for the affinity measurement to minimize the avidity effects caused by the bivalent nature of the antibodies. Human antibody capture kit (Cytiva) was used to immobilize Anti-human IgG (Fc) antibody to a CM5 sensor chip (Cytiva) by amine coupling at approximately 6000 RU. The sample antibody was captured at 62.5 ng/mL for 60 s at a flow rate of 10 μL/min, resulting in capture levels between 10 and 25 RU. A 4-fold dilution series of PCSK9 (Acro Biosystems) with concentrations ranging from 50 nM to 12 pM was flowed over the captured IgG surface for 240 s at a flow rate of 50 μL/min with a dissociation time of 1800 s. The surface was regenerated with 3M MgCl_2_ between measurement cycles. The association and dissociation values were determined with Biacore Insight Evaluation software using the Langmuir 1:1 global fitting procedure. All samples were run in duplicates.

### AC-SINS

AC-SINS was performed as described earlier (Geng et al., 2016). Briefly, 20 nm gold nanoparticles (Ted Pella) were washed and diluted by centrifuging 6 min at 21,130 x *g*, removing 95% of the supernatant and resuspending to 1.5 times of the original volume in Milli-Q water. The washed nanoparticles were conjugated overnight at room temperature (RT) with 160 μg/mL goat anti-human Fc capture antibody (Jackson ImmunoResearch Labs Cat# 109-001-008, RRID:AB_2337530), which was buffer exchanged twice into 20 mM potassium acetate pH 4.3 using 40 kDa MWCO Zeba spin desalting columns. The conjugates were blocked with 0.1 µM thiolated PEG (MW 2000; Sigma-Aldrich) by incubating at RT for 1 h. Unbound anti-Fc antibody and excess thiolated PEG was washed by centrifugation, removing 95% of the supernatant and resuspending to 50% of the original volume in 2 mM potassium acetate pH 4.3. Next, the SEC-purified sample antibodies were diluted to 10 μg/mL using PBS with 100 μg/mL goat non-specific antibody (Jackson ImmunoResearch Labs Cat# 005-000-003, RRID:AB_2336985) added to mitigate non-specific binding. Next, 8 µL of the prepared capture conjugates were pipetted to wells of clear-bottomed 384-well plates (Fisher Scientific) and subsequently 72 µL of the prepared sample antibody was added and mixed by pipetting. After 2 h incubation, the absorbance spectra were measured using EnVision plate reader (Perkin Elmer) in 500–600 nm with 1 nm increments. A macro was used to fit a second order polynomial function to the measurement data to find the wavelength with the maximum absorbance value (plasmon wavelength). The plasmon wavelength of a blank sample with PBS was subtracted from the antibody sample values to determine the AC-SINS score. All measurements were made in triplicates.

### Polyreactivity ELISA

The polyreactivity ELISA protocol followed previously established procedures (Wardemann et al., 2003; Mouquet et al., 2010; Jain et al., 2017). Briefly, 50 µL of six different antigens: dsDNA (10 μg/mL, Sigma), ssDNA (10 μg/mL, Sigma), LPS (10 μg/mL, Sigma), KLH (10 μg/mL, Sigma), cardiolipin (10 μg/mL, Sigma) and insulin (5 μg/mL, Sigma) were coated onto 96-well ELISA plates (VWR) overnight at RT. The plates were washed three times with Milli-Q water, followed by blocking with 200 µL of ELISA buffer (PBS with 1 mM EDTA and 0.05% Tween-20) for 1–2 h at RT. After blocking, the plates were washed three times and 50 µL of the antibody samples were added at concentrations of 1,000, 250, 62.5 and 15.6 ng/mL in PBS and incubated for 2 h at RT, followed by three washes. HRP-conjugated goat anti-human IgG antibody (Jackson ImmunoResearch Labs Cat# 109-035-088, RRID:AB_2337584) was diluted 1:1,000 in ELISA buffer and 50 µL of it was added to each well for 1 h, followed by three washes. The plates were then blocked with ELISA buffer for 5 min, washed three times and 100 µL of TMB substrate (Thermo Scientific) was added according to the manufacturer’s instructions. After 15 min incubation at RT, 100 µL of 2M HCl was added to the wells to stop the reaction and absorbance at 450 nm was measured with EnVision plate reader. The absorbance of a blank sample with PBS was subtracted from the antibody samples. The polyreactivity score was calculated by taking an average of the PBS subtracted signals from all 4 concentrations of the sample antibody in all 6 antigens.

### Tm measurements by nanoDSF and measurement of polydispersity index by DLS

All DLS and nanoDSF measurements were performed using the Nanotemper Prometheus Panta instrument. Antibody samples (∼10 μL at 0.5–1 mg/mL) were loaded by capillarity into standard grade nano-DSF capillaries and placed on the Prometheus capillary holder. The DLS data for the samples was measured at 25°C, which allowed the determination of polydispersity index (PDI). Afterwards, the samples were subjected to a temperature ramping of 1°C/min from 20°C to 95°C. The melting temperature (Tm) values were obtained by monitoring the intrinsic tryptophan and tyrosine fluorescence at the emission wavelengths of 330 nm and 350 nm. To generate an unfolding curve, the ratio of the fluorescence intensities (F350 nm/F330 nm) was plotted vs. temperature. The thermal stability of a sample was described by the thermal unfolding transition midpoint Tm (°C), at which half of the protein population is unfolded. The Tm corresponds to the inflection point of the unfolding curve and was determined *via* the derivative of the curve.

### Analytical hydrophobic interaction chromatography

Antibody samples were diluted to 0.5–1 mg/mL with mobile phase A solution (1.5 M ammonium sulphate and 20 mM Tris, pH 7.0) to achieve a final ammonium sulfate concentration of 0.5 M before analysis. Two to 4 μg of the diluted antibody was loaded to a TSKgel Butyl-NPR, 2.5 µM, 4.6 mm × 3.5 cm column (Tosoh Bioscience) with a linear gradient of mobile phase A and mobile phase B solution (20 mM Tris, pH 7.0) over 10 min with 0.8 mL/min flow rate starting from 5% B and ending at 95% B. Peak retention time was monitored with UV absorbance at 220 nm using 1,260 Infinity II Bio-inert LC System (Agilent).

### *In silico* analysis and statistical analysis

The *in silico* analysis was done using the antibody structure prediction and protein descriptor calculations with Schrödinger BioLuminate using the variable domain sequences as input. Fifty-five most relevant parameters were chosen from the protein descriptors, which were related to the antibody’s charge, isoelectric point, AggScore, electrostatic patch energy and hydrophobic patch energy for each CDR separately. Similar structure prediction and protein descriptor calculations were done for 48 approved antibodies from the study by Jain et al. (Jain et al., 2017) and their average values were calculated to determine cut-off values for each parameter. The cut-off for each parameter was defined as the average score plus standard deviation of the modelled approved therapeutic antibodies. Statistical analysis was performed using GraphPad Prism 9.

## Data Availability

The datasets presented in this study can be found in online repositories. The names of the repository/repositories and accession number(s) can be found below: https://www.ncbi.nlm.nih.gov/, PRJNA937421.

## Supplementary material

SUPPLEMENTARY FIGURE S1 Determination of optimal pBxb1-EV to pBxb1-TV-GFP ratio for the highest stable integration efficiency in suspension adapted CHO-LP cells, measured 8 dpt without drug selection. The optimal vector ratio was determined to be 1:4.

SUPPLEMENTARY FIGURE S2 Demonstration of the high specificity of the Bxb1 RMCE system. **(A)** Fluorescent microscopy image of CHO-LP cells co-transfected with a mixture of BFP1.2, EGFP and dsRed encoding genes in pBxb1-TV and pBxb1-EV after 8 days under drug selection. **(B)** Flow cytometry analysis of CHO-LP cells co-transfected with a mixture of BFP1.2 and dsRed encoding genes in pBxb1-TV and pBxb1-EV after 10 days under drug selection.

SUPPLEMENTARY FIGURE S3 Representative sensorgrams of six bococizumab variants from affinity measurements using Biacore 8K+. Due to the slow off-rate of bococizumab, the dissociation was set to 30 minutes.

## Abbreviations

AC-SINS, Affinity Capture Self-Interaction Nanoparticle Spectroscopy; APC, Allophycocyanin; CDR, Complementary Determining Region; CHO, Chinese Hamster Ovary; CRISPR, Clustered Regularly-Interspaced Short Palindromic Repeats; DLS, Dynamic Light Scattering; Dpt, Days Post Transfection; DSF, Differential Scanning Fluorimetry; ELISA, Enzyme-Linked Immuno Sorbent Assay; FACS, Fluorescence Activated Cell Sorting; FRT, Flp Recombinase Target; IgG, Immunoglobulin G; mAb, Monoclonal Antibody; MACS, Magnetic Activated Cell Sorting; NGS, Next Generation Sequencing; PCSK9, Proprotein Convertase Substilisin/Kexin Type 9; PE, Phycoerythrin; SEC, Size-Exclusion Chromatography; SPR, Surface Plasmon Resonance; TALEN, Transcription Activator-Like Effector Nuclease; V_H_, Variable Heavy Region; V_L_, Variable Light Region.
