# Supplementary figures and images for: Selection of biophysically favorable antibody variants using a modified Flp-In CHO mammalian display platform
Source: Front Bioeng Biotechnol. 2023 May 9;11:1170081. doi: 10.3389/fbioe.2023.1170081 (PMC10203562; doi:10.3389/fbioe.2023.1170081)

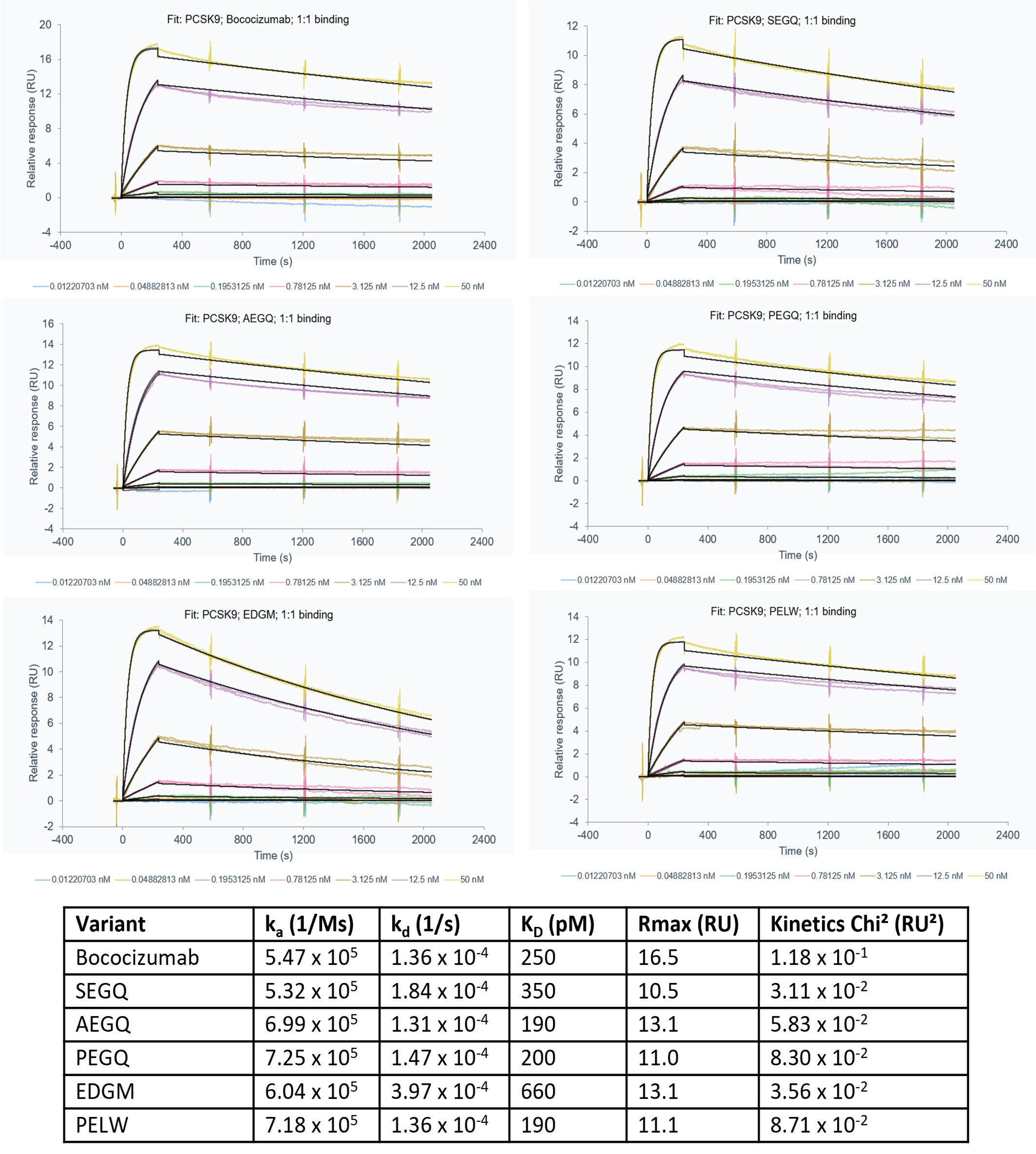

Supplement: Supplementary file 1 [file Image3.TIFF]

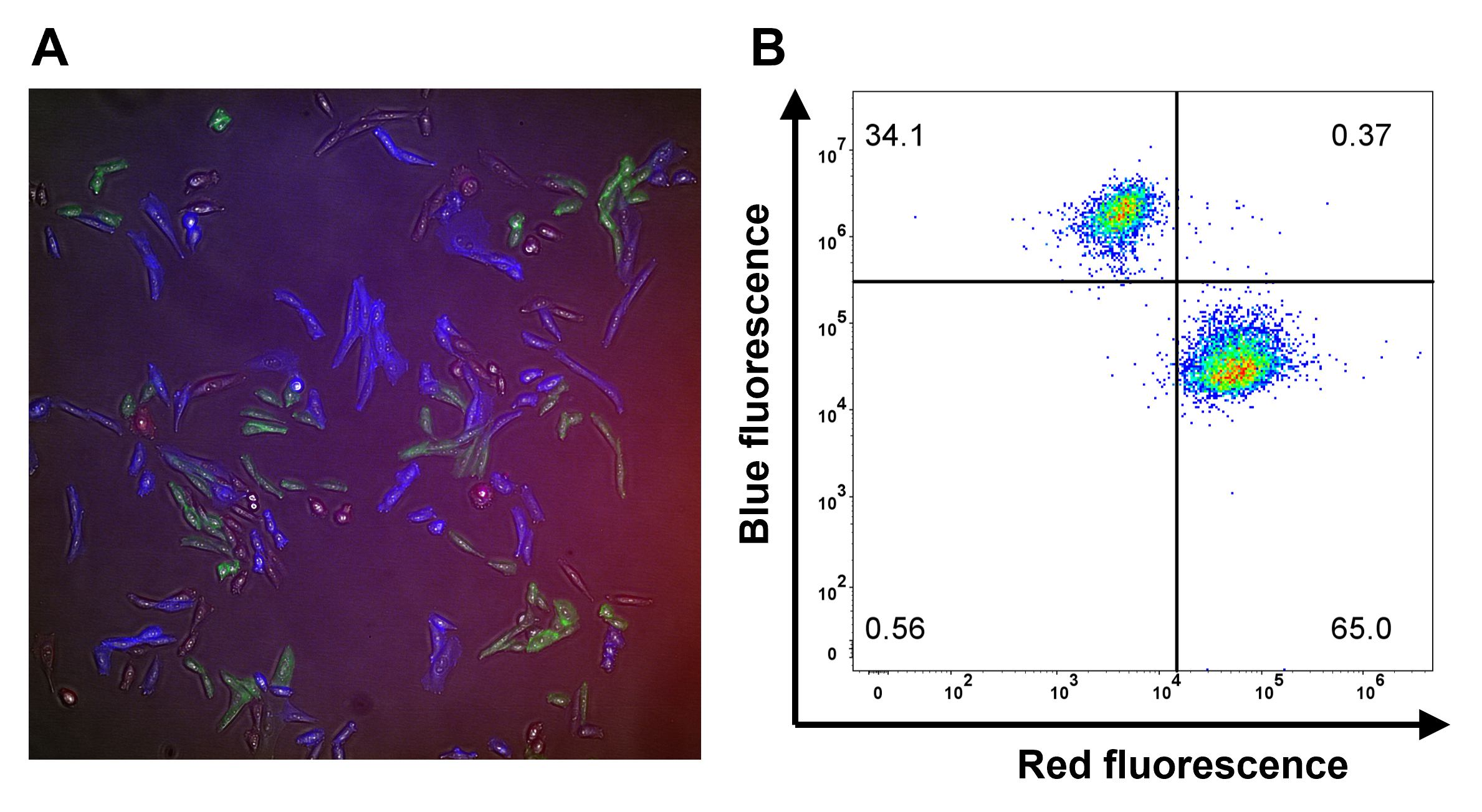

Supplement: Supplementary file 2 [file Image2.TIF]

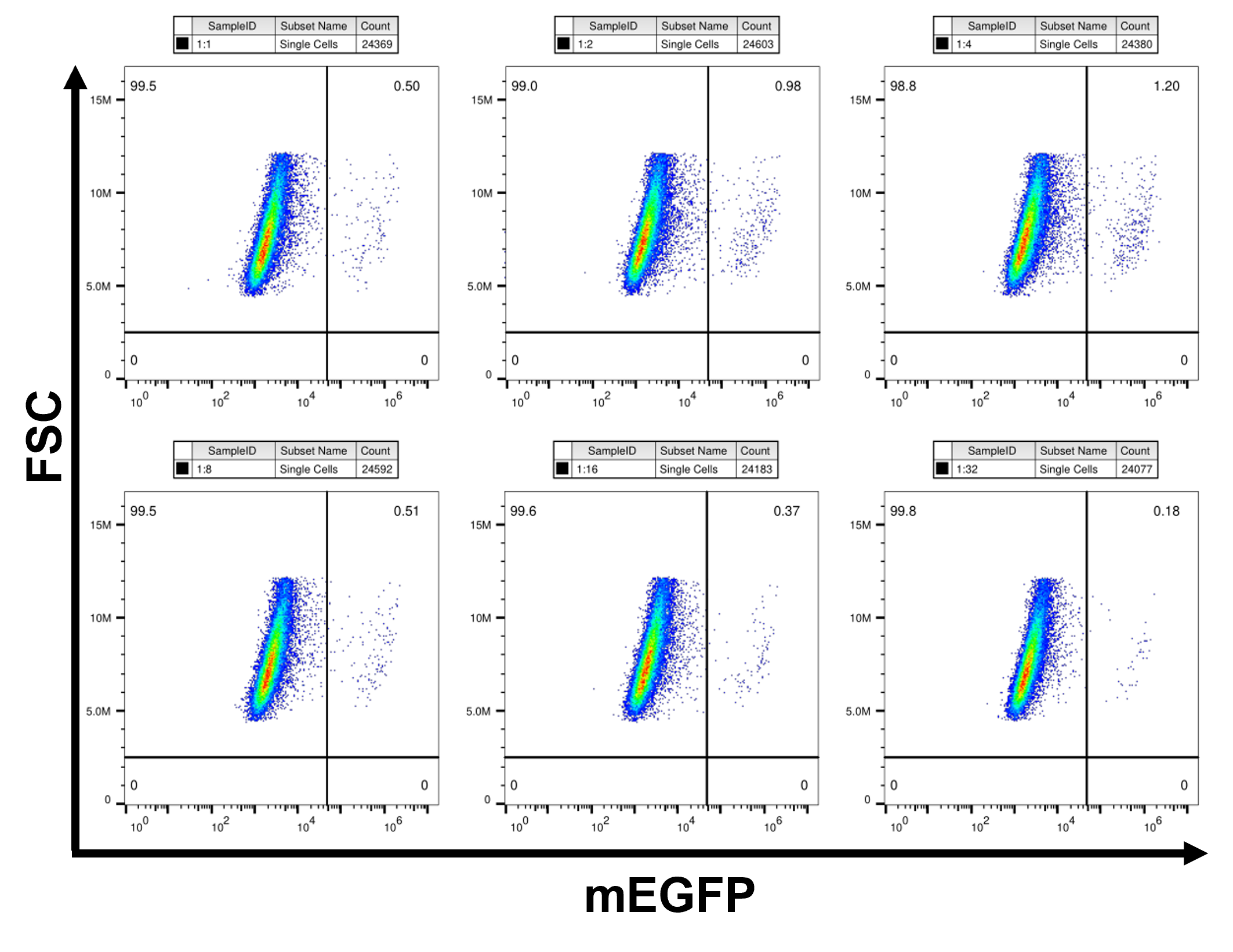

Supplement: Supplementary file 3 [file Image1.TIF]
